# The Directly Observed Therapy Short-Course (DOTS) strategy in Samara Oblast, Russian Federation

**DOI:** 10.1186/1465-9921-7-44

**Published:** 2006-03-23

**Authors:** Y Balabanova, F Drobniewski, I Fedorin, S Zakharova, V Nikolayevskyy, R Atun, R Coker

**Affiliations:** 1 HPA Mycobacterium Reference Unit, Clinical TB and HIV Group, St Bartholomew and Queen Mary School of Medicine, 2 Newark street, London E1 2AT, UK; 2 Samara Oblast Tuberculosis Dispensary, Samara, 154 Novo-Sadovaya Street, 443068, Russia; 3 Samara City Tuberculosis Dispensary N1, Pionerskaya street, Samara, 443001, Russia; 4 Center for Health Management, Tanaka Business School, Imperial College London, South Kensington campus, London SW7 2AZ, UK; 5 Department of Public Health and Policy, London School of Hygiene and Tropical Medicine, Keppel Street, London WC1E 7HT, UK

## Abstract

**Background:**

The World Health Organisation (WHO) defines Russia as one of the 22 highest-burden countries for tuberculosis (TB). The WHO Directly Observed Treatment Short Course (DOTS) strategy employing a standardised treatment for 6 months produces the highest cure rates for drug sensitive TB. The Russian TB service traditionally employed individualised treatment.

The purpose of this study was to implement a DOTS programme in the civilian and prison sectors of Samara Region of Russia, describe the clinical features and outcomes of recruited patients, determine the proportion of individuals in the cohorts who were infected with drug resistant TB, the degree to which resistance was attributed to the Beijing TB strain family and establish risk factors for drug resistance.

**Methods:**

prospective study

**Results:**

2,099 patients were recruited overall. Treatment outcomes were analysed for patients recruited up to the third quarter of 2003 (n = 920). 75.3% of patients were successfully treated. Unsuccessful outcomes occurred in 7.3% of cases; 3.6% of patients died during treatment, with a significantly higher proportion of smear-positive cases dying compared to smear-negative cases. 14.0% were lost and transferred out. A high proportion of new cases (948 sequential culture-proven TB cases) had tuberculosis that was resistant to first-line drugs; (24.9% isoniazid resistant; 20.3% rifampicin resistant; 17.3% multidrug resistant tuberculosis). Molecular epidemiological analysis demonstrated that half of all isolated strains (50.7%; 375/740) belonged to the Beijing family. Drug resistance including MDR TB was strongly associated with infection with the Beijing strain (for MDR TB, 35.2% in Beijing strains versus 9.5% in non-Beijing strains, OR-5.2. Risk factors for multidrug resistant tuberculosis were: being a prisoner (OR 4.4), having a relapse of tuberculosis (OR 3.5), being infected with a Beijing family TB strain (OR 6.5) and having an unsuccessful outcome from treatment (OR 5.0).

**Conclusion:**

The implementation of DOTS in Samara, Russia, was feasible and successful. Drug resistant tuberculosis rates in new cases were high and challenge successful outcomes from a conventional DOTS programme alone.

## Background

Since the 1990s the World Health Organization Directly Observed Therapy Short Course (DOTS) management strategy has become the internationally recommended approach for tuberculosis (TB) control programmes [1-3]. By the beginning of the new Millennium, 149 countries in the world had adopted the DOTS strategy to varying degrees and important measures of DOTS success (case detection and treatment success) were included in the Millennium Development Goals framework [4]. In the former Soviet Union (FSU) only a limited number of WHO DOTS implementation programmes exist and currently countries of the FSU report the lowest case detection rates (22%) with 9% of cases failing treatment and a death rate of 7% during treatment [4]. WHO has acknowledged that until TB is controlled in Africa and Eastern Europe, this disease will remain of major world-wide concern; current analysis indicates that it is unlikely that the Millennium Development Targets for TB will be met in these regions [4].

Russia is one of 22 TB high-burden countries as defined by the WHO [5,6]. Russia has a highly-specialised tuberculosis health care system with a large organisationally-vertical network of specialized institutes, dispensaries, hospitals, outpatient clinics, sanatoria and rural feldsher points. Case detection is based largely on the presence of radiological abnormalities on chest X-rays with or without bacteriological confirmation detected through a national policy of compulsory annual fluorographic population screening [7-9]. In contrast to the WHO recommended tuberculosis control DOTS strategy, which favour minimising hospital stays, clinical guidelines and health system financing incentives, TB patients in Russia experience frequent and lengthy hospitalisations, and historically have received individualised treatment regimens with doses of the main first line drugs and duration of chemotherapy varying from internationally accepted standard treatment regimens. The system also included prolonged periods of follow-up and repetitive courses of anti-relapse therapy [10,11].

The rationale for implementing the DOTS strategy in Russia is to establish cost-effective tuberculosis control by reducing unnecessary care costs due to lengthy hospitalisations, while improving cure rates and reducing the development of drug resistant TB [3,7,12-14].

In 2002, with assistance from the UK Department for International Development, a TB control programme that adhered to internationally accepted norms and standards was launched by the regional Ministry of Health. We have reported elsewhere on the considerable body of research undertaken in Samara that explores the epidemiological profile, the health care system structures and processes, and public health challenges being faced by the oblast [7,9-12], [15-23]

This paper describes the clinical features and outcomes of patients recruited to a DOTS programme which was implemented in civilian and prison sectors in Samara Oblast.

## Methods

At the initial stage of implementation of DOTS a standard protocol was agreed with the Regional Ministry of Health. This was followed by extensive training of medical doctors and TB nurses with the involvement of WHO and experts from Russian Federal TB Institutes. Two project medical co-ordinators based in Samara were appointed to oversee implementation which was rolled out in three phases.

Under phase one, initiated in April 2002, patient recruitment commenced at two pilot TB dispensaries in Samara City and at two TB prison colonies (one, an inpatient prison facility used for initial therapy, the second an outpatient facility where continuation of therapy occurred) that looked after all prisoners with TB in the oblast. Recruitment was expanded in January 2003, under phase two, to all TB facilities in Samara city (five dispensaries and three TB hospitals) and to the neighbouring city Togliatti. Under phase three, a further rollout occurred in January 2004 to the rural district of Krasny Yar. We report results through all three phases and include patients recruited up to the third quarter of 2004.

Patients were recruited into standard WHO categories (Table 1). In 2002, initially only new cases were recruited (category I and III). From April 2003, recruitment was extended to include relapse cases (category II). Because of the prevalence of drug resistance and concerns that resistance profiles would be further amplified [23-25] chronic cases were ineligible for recruitment.

**Table 1:** WHO treatment categories and outcome definitions [28]

**Category**	**Description**
**I**	new cases of smear- positive pulmonary tuberculosis and other newly diagnosed seriously ill patients with severe forms of tuberculosis (i.e. disseminated tuberculosis, tuberculous meningitis, tuberculosis spondyolitis with neurological complications, tuberculosis pericarditis, peritonitis, bilateral or extensive pleurisy, smear-negative pulmonary tuberculosis with extensive parenchymal involvement, intestinal tuberculosis, genito-urinary tuberculosis, etc.)
**II**	relapse and failure patients, those who interrupted treatment, and "other" patients who were previously treated for more than 1 month not under a DOTS treatment program
**III**	new cases of smear-negative pulmonary tuberculosis and extra-pulmonary tuberculosis

**Outcomes**	
**Cure**	Patients are considered as cured if his/her smear/culture was positive before the onset of treatment, if they have completed a course of anti-tuberculosis chemotherapy and their smear/culture is negative at 5 or more months of treatment and at the end of treatment.
Treatment completed*	Patients who were smear and culture negative before the onset of treatment and thereafter, and have completed a full course of treatment. Patients who were smear and/or culture positive before the onset of treatment and have completed a full course of anti-tuberculosis chemotherapy but failed to have the required number of negative smears and/or cultures.
**Treatment failure**	A patient who failed to achieve bacteriological conversion within *5 (FIVE) *months after the start of treatment, or, after previous conversion, becomes sputum smear or culture-positive again. Also a patient who was initially smear-negative before starting treatment and became smear-positive after competing the initial phase of treatment.
**Death**	Patient who dies for any reason during the course of treatment.
**Default (interruption)**	Patient whose treatment was interrupted for two consecutive months or more.

**Transfer out**	Patient who has been transferred before the completion of his/her treatment to another recording and reporting unit and for whom the treatment outcome is not known.

* Treatment success is defined as the sum of patients cured and those who have completed treatment.

Given implementation of the internationally supported programme ceased in third quarter 2004, clinical outcomes presented are until the third quarter of 2003. Outcomes for patients recruited subsequently were registered within the newly adopted Russian national system which continued following this programme [26,27].

Standard TB control treatment outcomes were recorded (Table 1). Treatment success under the DOTS strategy was determined by cures and treatment completions and unsuccessful treatment included patients who failed and defaulted [28].

A modified feature of the programme was introduced where patients registered initially under the DOTS cohort could be transferred to an "individual treatment regimen", an approach that reflected the Russian legacy of individualised approaches to treatment. According to the prevailing views of Samara phthisiatrists not only patients who were diagnosed with MDRTB but also some severely ill patients or those with severe co-morbidities or perceived adverse reactions would be removed from the DOTS programme and managed within the regional TB programme using an individualised approach in line withy earlier Russian traditions. Cases, following recruitment, which were subsequently determined to have MDR TB, were transferred out to an individually-tailored MDRTB drug regimen. A further feature of the modification of the DOTS programme was the continuation of the intensive phase of treatment beyond two months (for one more month) despite patients becoming smear-negative in the end of the second month of therapy intensive phase. This was done, in accordance with Russian traditions, where extensive radiological changes were present.

Standard technical approaches to documentation and diagnostic/treatment protocols were employed[28]. Sputum collection was performed at recommended intervals. During the intensive phase of therapy ethambutol was administered instead of streptomycin because a previous drug resistance survey had documented very high rates of primary resistance to streptomycin [9,23].

For all patients smear microscopy and culture was performed at recommended intervals. Smear microscopy and culture were performed using standard Ziehl-Neilsen microscopy and culture on Lowenstein-Jensen media. All positive isolates were tested for drug susceptibility to isoniazid, rifampicin, ethambutol and streptomycin. Quality-assured drug susceptibility testing (DST) was performed at three civil and one prison site using an absolute concentration method on Lowenstein-Jensen media. DST was assured by a period of training by staff from the WHO Supranational Reference Laboratory (SRL) in London (Health Protection Agency MRU) and in Samara. A blinded analysis of a test panel of TB cultures was performed. A proportion (10%) were retested by the SRL in London.

DNA was extracted and Beijing family strains were analysed in London and Samara by detection of the IS6110 insertions in the *dnaA-dnaN *intergenic region on a proportion of sequential isolates (n = 740).

Direct supervision of treatment adherence was completed by TB nurses at TB hospitals and dispensaries with out-patients receiving treatment daily or three times weekly. Upon release, ex-prisoners completed their treatment upon transfer of their care to the civilian service.

Medical co-ordinators performed regular visits to all participating DOTS sites to support implementation, ensure recruitment was maintained, and review documentation and adherence to the protocol. Over-arching project management group meetings which included all clinical stakeholders and the project directors from each DOTS site occurred on a monthly basis.

Socially disadvantaged patients were identified by a responsible physician at each dispensary and offered additional support to encourage treatment adherence with weekly food packages at a cost of 100 Russian Roubles (3 Euros) per person per week.

Data were entered and stored into a password protected database. The statistical analysis was performed using Excel and SPSS 12. Proportions with 95% confidence intervals (CI), relative risks (RR), odds ratios (OR), and χ^2 ^test are used for comparison of categorical variables.

The study was approved by the Samara Regional Ethics Committee.

## Results

2,099 patients were recruited from 1^st ^April 2002 to 30^th ^September 2004, including 1,971 individuals with pulmonary tuberculosis (93.9%) and 128 patients with extrapulmonary disease (6.1%); 1,684 of recruits were men (80.2%) and 415 (19.8%) women. 640 patients were recruited in the prison sector and 1459 were civilian TB patients.

One third (33.1%; 694/2,099) of recruited patients were WHO category I and 24.3% (162/694) of these were smear-negative cases with extensive parenchymal involvement; 58.8% (1,234/2,099) of cases were WHO category III patients. Recruitment into WHO category II was limited to relapse cases only and 171 patients (8.1%) were recruited.

The mean age of patients was 38.5 years (95%CI 37.9–39.1 years; range: 16–90 years) with prisoners being significantly younger than civilians (mean age 30.9 years; 95%CI 30.2–31.6 years versus mean age 41.9 years; 95%CI 41.1–42.7 years). Female patients were older than male patients (mean age of men was 38.0 (95%CI 37.4–38.6) years and mean age of women with TB was 40.4 (95%CI 38.8–42.0) years).

Details of bacteriologically (smear and/or culture) confirmed cases are shown in Table 2. Table 3 shows differences between the infectious status of civilian and prison populations with TB where civilians were more likely to have infectious disease whether determined by smear status or culture status. Overall the rate of laboratory diagnosed TB cases was slightly higher in civilian patients than prisoners.

**Table 2:** Proportion of bacteriologically confirmed new and relapse pulmonary cases (II quarter 2002 – III quarter 2004)

	**Microscopy**			**Culture**		
	
	**Total No of cases**	**Smear positive**	**Smear negative-**	**Total No of cases**	**Culture positive**	**Culture negative**
***New cases***						
**Civil**	1227	462 (37.7%)	765 (62.3%)	1341	764 (57.0%)	577 (43.0%)
**Prison**	586	70 (11.9%)	516 (88.1%)	587	184 (31.3%)	403 (68.7%)
**Total**	1813	532 (29.3%)	1281(70.7%)	1928	948 (49.2%)	980 (50.8%)
***Relapses***						
**Civil**	101	39 (38.6%)	62 (61.4%)	118	76 (64.4%)	42 (35.6%)
**Prison**	51	5 (9.8%)	46 (90.2%)	53	18 (34.0%)	35 (66.0%)
**Total**	152	44 (28.9%)	108 (71.1%)	171	94 (55.0%)	77 (45.0%)
***Total sample (new and relapse cases)***						
**Civil**	1328	501 (37.7%)	827 (62.3%)	1459	840 (57.6%)	619 (42.4%)
**Prison**	637	75 (11.8%)	562 (88.2%)	640	202 (31.6%)	438 (68.4%)
**Total**	1965	576 (29.3%)	1389 (70.7%)	2099	1042 (49.6%)	1057 (50.4%)

*a proportion of patients could not expectorate a sputum sample of a quality that would be suitable for culturing

**Table 3:** Difference in infectious status between civilians and prisoners

	**Civil (n positive/total tested, %)**	**Prison (n positive/total tested, %)**	**OR (95%CI)**	**RR (95%CI)**
***New cases***				
Smear+ *	462/1227 (37.7%)	70/586 (11.9%)	4.5 (3.4–5.9)	3.2 (2.5–4.0)
Culture+ *	764/1341 (57.0%)	184/587 (31.3%)	2.9 (2.4–3.6)	1. 8 (1.6–2.1)
***Relapses***				
Smear+ *	39/101 (38.6%)	5/51 (9.8%)	5.8 (2.1–15.8)	3.9 (1.7–9.4)
Culture+ *	76/118 (64.4%)	18/53 (34.0%)	3.5 (1.8–7.0)	1.9 (1.3–2.8)
***Total sample (new and relapse cases)***				
Smear+ *	501/1328 (37.7%)	75/637 (11.8%)	4.5 (3.5–5.9)	3.2 (2.6–4.0)
Culture+ *	840/1459 (57.6%)	202/640 (31.6%)	2.9 (2.4–3.6)	1.8 (1.6–2.1)

* statistically significant at p < 0.05

**Figure 1:**
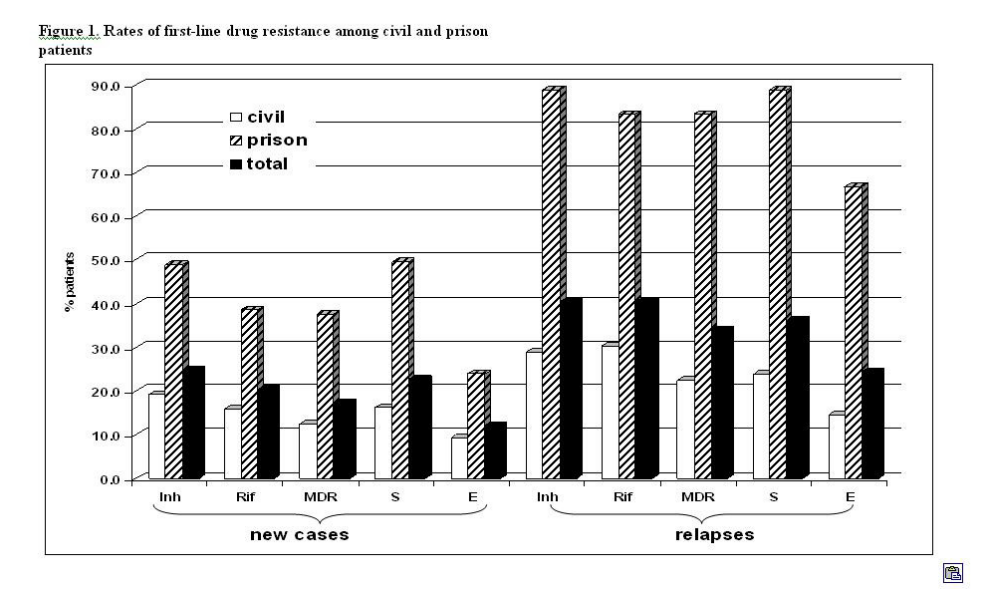
Rates of first-line drug resistance among civil and prison patients.

Cultures from 948 sequentially new and 94 relapse cases were isolated and tested for susceptibility to first-line drugs. Of the new cases, 24.9% (236/948) new cases had isolates resistant to isoniazid, 20.3% (192/948) new cases had isolates resistance to rifampicin, and 17.3% (164/948) had MDRTB (vs 34.0% (32/94) of relapse cases being MDR (OR-2.5; 95%CI 1.6–3.9). Table 3 and Figure 1 show the differences between civilian and prison patients.

Molecular epidemiological analysis demonstrated that a half of all isolated strains (50.7%; 375/740) belonged to the Beijing family. Of note, seven isolates (0.9%) were mixed strains. The prevalence of the Beijing strain (60.9%; 117/192) among prisoners was significantly higher (OR-1.7; 95% CI 1.2–2.4) than in civilians (47.1%; 258/548) confirming earlier research findings in a drug resistance survey in the same region in the preceding year [19].

For 709 isolates data on both drug resistance and strain type were available (31 isolates were non-viable or were contaminated and DST could not be performed). Drug resistance including MDR TB was strongly associated with being infected with the Beijing strain (for MDR TB 35.2% in Beijing strains versus 9.5% in non-Beijing strains, OR-5.2 (3.4–7.9) (Table 5) confirming earlier research in a different population of patients treated under the Russian system in the same region[19].

**Table 4:** Difference in resistance rates between civil and prison patients

***New cases***	**Prison and civilian (N-948)**	**Prison (N-184)**	**Civil (N-764)**	**OR (95%CI)**	**RR (95%CI)**
Inh*	236 (24.9%)	90 (48.9%)	146 (19.1%)	3.6 (2.5–5.1)	2.6 (2.0–3.2)
Rif*	192 (20.3%)	71 (38.6%)	121 (15.8%)	3.0 (2.1–4.2)	2.4 (1.9–3.1)
MDR TB*	164 (17.3%)	69 (37.5%)	95 (12.4%)	3.8 (2.6–5.5)	3.0 (2.3–3.9)
S*	215 (22.7%)	91 (49.5%)	124 (16.2%)	4.5 (3.2–6.4)	3.0 (2.5–3.8)
E*	115 (12.1%)	44 (23.9%)	71 (9.3%)	2.7 (1.8–4.2)	2.6 (1.8–3.6)

***Relapses ***	**Prison and civilian (N- 94)**	**Prison (N-18)**	**Civil (N-76)**	**OR (95%CI)**	**RR (95%CI)**

Inh*	38 (40.4%)	16 (88.9%)	22 (28.9%)	16.4 (3.5–77.6)	3.1 (2.1–4.5)
Rif*	38 (40.4%)	15 (83.3%)	23 (30.3%)	10.0 (2.5–36.5)	2.8 (1.9–4.10
MDR TB*	32 (34.0%)	15 (83.3%)	17 (22.4%)	14.7 (3.8–57.1)	3.7 (2.3–5.9)
S*	34 (36.2%)	16 (88.9%)	18 (23.7%)	21.8 (4.5–104.3)	3.8 (2.4–5.8)
E*	23 (24.5%)	12 (66.7%)	11 (14.5%)	10.2 (3.1–32.9)	4.6 (2.4–8.7)

*statistically significant at p < 0.05

**Table 5:** Comparison of first-line resistance levels in Beijing compared to non-Beijing strains (n = 709^)

**Drug**	**Beijing strain (n = 361)**		**Non-Beijing strain (n = 348)**		**OR (95%CI)**	**RR (95%CI)**	**RD (95%CI)**
				
	*N resistant*	*% resistant (95%CI)*	*N resistant*	*% resistant (95%CI)*			
Isoniazid*	170	47.1 (48.0–52.3)	53	15.2 (11.7–19.3)	5.0 (3.5–7.1)	3.1 (2.4–4.1)	31.9 (25.2–38.5)
Rifampicin*	140	38.8 (33.9–43.9)	41	11.8 (8.7–15.5)	4.7 (3.2–7.0)	3.3 (2.4–4.5)	27.0 (20.7–33.3)
MDR TB*	127	35.2 (30.4–40.2)	33	9.5 (6.7–12.9)	5.2 (3.4–7.9)	3.7 (2.6–5.3)	25.7 (19.6–31.8)
Streptomycin*	144	39.9 (34.9–45.0)	45	12.9 (9.7–16.8)	4.5 (3.1–6.5)	3.1 (2.3–4.2)	27.0 (20.5–33.4)
Ethambutol*	89	24.7 (20.4–29.3)	26	7.5 (5.0–10.6)	4.1 (2.5–6.5)	3.3 (2.2–5.0)	17.2 (11.7–22.)

* statistically significant difference at p < 0.001

^ both epidemiological and drug resistance results were available for 709/1042 (68.0%) cultures; 31 cultures were non viable or contaminated; cultures were drawn from all dispensaries and prison facilities in Samara City.

Multivariate analysis suggests that being a prisoner (OR – 4.4; 95%CI 2.7–7.1), having a relapse of TB (OR-3.5; 95%CI 1.7–7.1), being infected with the Beijing family strains (OR-6.5; 95%CI 4.0–10.5) and having unsuccessful outcome of treatment (OR-5.0; 95%CI 1.1–22.7) were risk factors for MDR TB.

During the course of treatment the majority (97.7%; 284/290) of smear-positive new cases converted by the end of the intensive phase of treatment.

Treatment outcomes among new cases confirmed by culture are shown in Table 6. Because recruitment of relapses was initiated at a later stage, the number of these is small. Overall 85.4% (786/920) of newly diagnosed and recruited patients were treated according to the WHO protocol. Nearly fifteen percent (134/920) of patients were transferred out of the DOTS clinical protocol and this included patients transferred to individual regimens because MDRTB (17.3% of all new cases were MDR and 34.0% of all relapse cases) or extensive radiological abnormalities, adverse drug reactions, or co-morbidities). MDR TB patients were removed from the programme according to DOTS project criteria and further treated with tailored schemes using second-line drugs. More smear positive patients were transferred out than smear-negative cases (22.4% versus 11.6%; OR-2.2; 95%CI 1.5–3.2). In total 75.3% (592/786) of patients were successfully treated and in 7.3% (57/786) treatment failed or patients defaulted. The odds of failing treatment or defaulting were higher in smear positive patients (OR – 10.6; 95% CI 3.4–32.8).

**Table 6:** Cohort outcomes for new cases confirmed by culture

	**Total registered**	Total remaining in cohorts** n, %	Excluded from cohorts* n, %	**Cured n, %**	**Treatment completed n, %**	**Successful treatment n,% ***rows e+f*	**Failure n, %**	**Default n, %**	**Unsuccessful treatment n, % ***rows h+i*	**Died n, %**	**Lost and transferred out n, %**
*a*	*b*	*c*	*d*	*e*	*f*	*g*	*h*	*i*	*j*	*k*	*l*

**Civil**	**569**	**467 (82.1)**	**102(17.9)**	**106 (22.7)**	**263(56.3)**	**369(79.0%)**	**12(2.6)**	**36 (7.7)**	**48(10.3)**	**28 (6.0)**	**22 (4.7)**
*Smear+*	207	161 (77.8)	46 (22.2)	106 (65.8)	0 (0.0)	106 (65.8%)	11 (6.8)	16 (9.9)	27 (16.8)	22 (13.7)	6 (3.7)
*Smear-*	362	306 (84.5)	56 (15.5)	0 (0.0)	263 (85.9)	263 (85.9%)	1 (0.3)	20 (6.5)	21 (6.9)	6 (2.0)	16 (5.2)
*Culture+*	---	266	---	95 (35.7)	103 (38.7)	198 (74.4%)	11 (4.1)	23 (8.6)	34 (12.8)	25 (9.4)	9 (3.4)
*Culture-*	---	201	---	11 (5.5)	160 (79.6)	171(85.1%)	1 (0.5)	13 (6.5)	14 (7.0)	3 (1.5)	13 (6.5)
**Prison**	**351**	**319 (90.9)**	**32 (9.1)**	**5 (1.6)**	**218(68.3)**	**223(69.9%)**	**5 (1.6)**	**3 (0.9)**	**8 (2.5)**	**0 (0.0)**	**88(27.6)**
*Smear+*	43	33 (76.7)	10 (23.3)	5 (15.2)	11 (33.3)	16 (48.5%)	2 (6.1)	0 (0.0)	2 (6.1)	0 (0.0)	15 (45.5)
*Smear-*	308	286 (92.9)	22 (7.1)	0 (0.0)	207 (72.4)	207 (72.4%)	3 (1.0)	3 (1.0)	6 (2.1)	0 (0.0)	73 (25.5)
*Culture+*	---	74	---	4 (5.4)	44 (59.5)	48 (64.9%)	3 (4.1)	0 (0.0)	3 (4.1)	0 (0.0)	23 (31.1)
*Culture-*	---	245	---	1 (0.4)	174 (71.0)	175 (71.4%)	2 (0.8)	3 (1.2)	5 (2.0)	0 (0.0)	65 (26.5)
**Total**	**920**	**786 (85.4)**	**134(14.6)**	**111 (14.1)**	**481(61.2)**	**592(75.3%)**	**17(2.2)**	**40 (5.1)**	**57 (7.3)**	**28 (3.6)**	**110(14.0)**
*Smear+*	250	194 (77.6)	56 (22.4)	111 (57.2)	11 (5.7)	122 (62.9%)	13(6.7)	16 (8.2)	29 (14.9)	22 (11.3)	21(10.8)
*Smear-*	670	592 (88.4)	78 (11.6)	0 (0.0)	470 (79.4)	470 (79.4%)	4 (0.7)	23 (3.9)	27 (4.6)	6 (1.0)	89(15.0)
*Culture+*	---	340	---	99 (29.1)	147 (43.2)	246 (72.4%)	14 (4.1)	23 (6.8)	37(10.9)	25 (7.4)	32 (9.4)
*Culture-*	---	446	---	12 (2.7)	334 (74.9)	346 (77.6%)	3 (0.7)	16 (3.6)	19 (4.3)	3 (0.7)	78 (17.5)

*a proportion of patients were excluded from cohort (transferred to a different regimen due to MDR TB, extensive radiological abnormalities, drug adverse reactions, severe accompanying pathology)

** N of patients remained in the cohort (column "total") is taken as a denominator

There was no statistically significant difference in treatment outcomes between male and female patients.

The rates of unsuccessful treatment was higher among civilians compared to prisoners (OR-4.5; 95%CI 2.1–10.0)

Twenty-eight patients (3.6 %; 28/786) died during the course of treatment with a significantly higher proportion (11.3%) of smear-positive cases dying versus smear-negative (OR -12.5; 95%CI 5.0–31.3).

## Discussion and conclusion

The WHO have argued that the introduction of DOTS cohort treatment strategies improves case detection and treatment and leads to a reduction in TB prevalence and death rates by cutting the duration of illness and case fatality.

Two examples from middle and high incidence countries (Peru and China) support this view. In Peru, the incidence rate of pulmonary TB has decreased annually by 6% after the nationwide implementation of DOTS[29]. In 13 provinces of China that implemented DOTS, the prevalence rate of culture-positive TB was cut by 30% between 1990 and 2000 [30]

The introduction of DOTS resulted in profound changes to the delivery of clinical care within the Samara TB Service. Although a direct observation component had, broadly. been present within the old system through lengthy hospitalisation periods, the strict adherence of physicians to standard regimens, the emphasis on laboratory diagnosis, and a robust system of recording and reporting of cohorts were new [7].

Similarly fewer than 70% of patients with TB were cured or completed treatment in Samara compared to 75.3% in the cohort groups. This is in keeping with the cure rates reported for DOTS programmes internationally (Table 7) and the global treatment success rate under DOTS has been high since the first observed cohort in 1994 (77%)[4].

**Table 7:** Comparison of Samara DOTS cohort with overall treatment success and outcome for global DOTS cohorts in 2002

%	Died	Failed	Defaulted	Treatment success
Samara, Russia	3.6	2.2	5.1	75.3
Eastern Europe	6.7	8.8	6.4	74.9
Central europe	5.0	3.5	6.0	79.7
East Mediterranean	3.2	1.4	7.8	83.5
Established markets	9.7	2.4	2.6	76.5
Latin America	4.3	1.2	6.0	83.4
Southeast asia	3.9	2.5	6.4	85.1
Western Pacific	2.2	0.9	2.2	89.1

Established markets include member states of the EU, North America, Australia, New Zealand and Japan.

The relatively high failure rates noted elsewhere in Eastern Europe, (9% of cases failed treatment and 7% died during treatment) are believed to be associated with high rates of multidrug resistance (which in itself is an indicator of a programme with low cure rates previously). In Samara, prior to the introduction of the DOTS cohort strategy we established that drug resistance was high in both new (approx 20%) and chronic cases in Samara [9,17,23].

Dye et al [4] further established that the prevalence will decrease sooner if case detection by DOTS programs (and hence the quality of treatment) can be improved more quickly, thus reducing the burden of illness during this period in future years. The DOTS programmes emphasise the importance of bacteriological confirmation. [7]Prior to the establishment of the cohort, there were more than 1.5 million flurographic examinations of the general population for early diagnosis of TB reflecting the national policy of fluorography screening of the population for TB for early diagnosis. We have reported on the subjective nature of radiological examination elsewhere [31] and emphasised the need for bacteriological confirmation of the diagnosis in line with international standards.

Previously, less than one-third (30.1%) of cases were bacteriologically confirmed (Coker et al, 2003 IUATLD) compared to the DOTS cohort where 49.6% of all cases (and 57.6% in civilian cases) were bacteriologically confirmed. [10,11]Overall the proportion of cases which had a bacteriological confirmation of the diagnosis was similar to rates reported from other regions of Russia [4];.

[31]Although the laboratory component of the TB service in Samara Oblast has been extensively upgraded and improved with prison and civil laboratory services working to these improved standards, maintenance and further quality improvement remains a priority. Without appropriate laboratory support, over-diagnosis of tuberculosis remains a possibility, resulting in unnecessary treatment and side-effects without benefit, and compounding service inefficiencies [13].

Relatively low default rates occurred with implementation of DOTS in Samara. This may be, in part, attributable to a programme of externally financed social support. This component was discontinued after external funding ceased, and it remains to be seen whether adherence rates will suffer. Of note, substance abuse, alcoholism, poverty and unemployment are common amongst patients with TB in Samara Oblast, co-factors likely to influence treatment adherence [22]. The sustainable success of DOTS in Russia is likely to be dependent on how care and support for these social and behavioural factors are integrated into TB care systems.

Effective responses in support of TB control demand political commitment and investment from local and federal budgets into non-medical support to patients and their families. However, few integrated social support systems for tuberculosis patients currently exist, and current laws and regulations have the potential to ensure that health and social care budgets remain disconnected from each other and from need [32,33]. Consequently, to compensate for inadequate social support systems for tuberculosis patients, providers use sophisticated practices to ensure lengthy admissions in the winter months – a response to social rather than medical need [32,33]. Whilst the two recent decrees on TB control issued in 2003 (#109 and #50) [26,27] support convergence of Russian TB control practices with WHO's DOTS strategy (with some specific differences reflecting Russia's clinical legacy), the sustainability of reforms needed to ensure cost-effective implementation such that DOTS implementation is allied to structural reform remains uncertain.

The rate of successful treatment (75.3% overall and 79.0% in civil sector) though below the 85% WHO target, was higher than reported from other several DOTS pilot regions in the former Soviet Union (68.1% according to the meta-analysis performed by Faustini et al, 2005 [25]). The zero mortality among prisoners may be misleading: several patients died after data censoring. Furthermore, policy that very severely ill patients are released from prison for treatment in the civilian sector means that deaths of these ex-prisoners are recorded as civilian deaths.

The high prevalence of drug resistance and the frequency of the Beijing strain family (previously shown to be associated with drug resistance) [19] remains a major clinical and public health challenge. Extremely high rates of drug resistance among prisoners despite significantly lower default rates in prison likely reflects on-going transmission of resistant strains. Rapid isolation of MDR TB cases, good co-ordination between the prison and civilian TB services and enhancement of infection control and treatment are needed to prevent further nosocomial and institutional spread of MDR TB and would increase the success of the current TB programme. This issue is likely to become considerably more of a problem as the emergent epidemic of HIV in Samara matures

Although the DOTS strategy does not include specific therapy for multi-drug resistant cases its effective implementation reduces the occurrence and further transmission of resistant strains [34]. However, in regions such as Samara with very high rates of MDR TB it is essential to ensure the availability of appropriate and timely diagnosis and treatment of existing cases as well as preventing the development of new ones. Rapid drug susceptibility techniques, with appropriate treatment to be tailored to circumstance may be necessary. Cost-effectiveness analysis of rapid methods in the post-Soviet context is required to inform investment and policy changes.

## Abbreviations

CI – Confidence Interval

DOTS – Directly Observed Therapy Short-Course

MDR TB – multi-drug resistant tuberculosis

OR – Odds ratio

RR – Risk ratio

SRL – Supranational Reference Laboratory

TB – tuberculosis

WHO – World Health Organization

## Competing interests

The author(s) declare that they have no competing interests

## Authors' contributions

YB participated in the design of the study, its coordination, acquisition of data, statistical analysis and drafted the manuscript; RC participated in acquisition of funding, design of the study, its supervision and drafted the manuscript; IF participated in administration of the study, its design and revision of the manuscript; SZ participated in design and coordination of the study, data acquisition and manuscript revision; VN carried out laboratory work and revised the manuscript, RA participated in acquisition of funding, design of the study, its supervision and revision of the manuscript, FD participated in acquisition of funding, design of the study, its supervision and gave final approval of the version to be published. All authors read and approved the final manuscript.
